# Sustainable supplier selection based on VIKOR with single-valued neutrosophic sets

**DOI:** 10.1371/journal.pone.0290093

**Published:** 2023-09-14

**Authors:** Xiaochun Luo, Zilong Wang, Liguo Yang, Lin Lu, Song Hu

**Affiliations:** 1 College of Economics and Management, Nanjing University of Aeronautics and Astronautis, Nanjing, China; 2 School of Economics and Management, Guangxi Normal University, Guilin, China; 3 School of Business, Hohai University, Nanjing, China; Sichuan University, CHINA

## Abstract

Considering economic, environmental, and social issues, the sustainability of the supply chain has drawn considerable attention due to societal and environmental changes within the supply chain network. The strategic study of the entire supply chain process and maximizing an organization’s competitive advantage depend heavily on supplier selection based on sustainable indicators. Selecting sustainable suppliers for the supply chain is challenging since it is a multi-criteria decision-making (MCDM) problem with significant uncertainty in the decision-making process. This study uses the VlseKriterijumska Optimizacija I Kompromisno Resenje (VIKOR) technique and single-valued neutrosophic sets (SVNS) to deal with the challenge of choosing a sustainable supplier with insufficient information. This method reduces the influence of personal experience and preference on the final evaluation results and the problem of excessive individual regret caused by factor correlation and improves the consistency of evaluation results. Finally, the method’s success and adaptability are demonstrated by sensitivity analysis and additional comparison analysis, and the benefits and drawbacks of the suggested framework are examined. Compared to other approaches, it can assist decision-makers in communicating fuzzy and uncertain information, offering a perspective and approach for MCDM in the face of such situations, and helping them select suppliers of high caliber and who practice sustainable business practices.

## 1 Introduction

A number of issues, including global warming and natural disasters, have evolved as a result of the rapid development of economic globalization and industrialization [1]. Globalization will continuously bring various economic changes, making these circumstances almost challenging to stop. However, it is worth considering that the direction we should be taking should provide a healthy environment and sustainable economy with fewer harmful effects [2]. Supply chain management (SCM) has historically been used to a considerable extent to forward economic objectives at the expense of society and the environment. It is essential to include environmental and social issues in the SCM metrics due to current environmental and resource concerns and the general expectations of social subjects and stakeholders for sustainability and ecological responsibility [3]. In today’s globalized businesses, sustainability is seen as a source of organizational advantage, and any unfavorable effects on sustainability that upstream suppliers feel will pass it down to downstream industries. For instance, suppliers to fashion companies like H&M and Next release dangerous chemicals into rivers, substantially harming the business’s reputation and customers’ purchase intentions [4].

Therefore, when choosing suppliers, businesses take into account both the direct economic benefits that suppliers contribute at the commercial level as well as whether their sustainable growth can result in indirect economic benefits [5]. SSCM, or sustainable supply chain management, has drawn much interest from businesses and the industry [6] and evolved into the primary business strategy [7]. From a system and integration standpoint, SSCM is a valuable management technique for resolving the conflict between a company and its environment and is extensively used at the same time [8].

The foundation of SSCM and the core of supplier management are excellent sustainable suppliers [9–11]. As the input of the supply chain, suppliers play an essential role in products, services, organizations, and sustainable performance [12–14]. To integrate all supply chain processes, businesses need an effective SCM. Selecting and evaluating suppliers is the first stage, but this process takes time and resources [15]. By choosing the suppliers with the greatest potential, a firm can benefit from a number of benefits, optimize financial rewards, and reduce costs and risks [16]. IKEA, for instance, controls its supply chain using socially and environmentally responsible methods while expanding its market share and attaining corporate success [17].

Traditionally, the economic aspect is regarded as the primary basis of supplier selection [18]. In addition to economic factors, green image, social concern, and economic policy are forcing companies to integrate SSCM [19]. Therefore, in the sustainable supplier selection process, evaluating and selecting suitable suppliers is an arduous task [1]. Confusion commonly develops when choosing sustainable providers due to multiple constraints, a lack of knowledge, complicated human cognitive processes, and the issue’s inconsistency. Several multi-criteria decision-making (MCDM) models are offered to evaluate potential suppliers; nevertheless, accurate and reliable information is insufficient to represent real-world, natural problems [20,21]. Academics and professionals pay attention to SSC-related concerns because they call for creating a systematic process that simultaneously handles competing for quantifiable and non-quantifiable aspects. A rigorous decision-making process goes into choosing sustainable suppliers. The evaluation procedure requires numerous qualitative and quantitative markers involving inaccurate and unclear data. By improving various MCDM practices, where a set number of suppliers are assessed following the quantitative and qualitative criteria taken into account by MCDM procedures, people attempt to solve such issues.

The economic, environmental, and social factors are combined to create the evaluation criteria for sustainable suppliers, and AHP and entropy weight is used to calculate the total importance. By extending the VIKOR approach to SVNS, SVNS is used to simplify the process. The measurement formula is considered, and the "relative distance" is adopted. The effectiveness of the method is measured by sensitivity analysis and method comparison. This paper subsequently identified numerous objectives after thoroughly evaluating relevant studies as follows:

1. Determine sustainable supplier selection criteria and evaluation measures, and effectively identify and evaluate supplier qualification.
2. How to deal with the uncertainty and inconsistency of evaluation information.
3. Assess the eligibility weight of the measures and select the most likely sustainable supplier.
4. How to develop a useful tool to select the best sustainable suppliers.

The remainder of this paper is organized as follows. The pertinent research on sustainable suppliers and MCDM techniques are found in Section 2, while the approaches are covered in Section 3. The development of a sustainable supplier evaluation approach is also covered in section 4. The mathematical analysis in Section 5 thoroughly explains the chosen tactics. Sensitivity analysis, discussion, and technique comparison serve to validate the model’s accuracy and reliability. Section 6 concludes by summarizing the findings and suggesting a course for further investigation.

## 2 Literature review

The external environment of businesses is unstable because of ongoing changes in consumer behaviour, technological development, and political climate. For the long-term growth of companies, especially the supply chain, it is crucial to assess and choose sustainable suppliers in an uncertain environment. As a result, the decision-making capabilities of the supplier have long been a subject of interest for academic research. Identification, screening, evaluation, analysis, and contract signing with suppliers are all parts of the supplier selection process [22]. The solution to the dilemma of sustainable supplier decision-making lies in the identification and selection of criteria. In the early supplier selection research paradigm, academics considered conventional metrics like cost/price, quality, service level, delivery time, flexibility, and delivery [23]. With the ongoing evolution of the economy and society, the supplier selection process have changed significantly. Environmental considerations are typically overlooked when choosing suppliers in the traditional manner. Green suppliers become more visible in SCM as a result of rising customer awareness of environmental issues and stakeholder pressure on companies to take these issues into account throughout their supply chains [24,25]. Green suppliers have successfully solved the supply chain issue by taking ecological concerns into account, however this approach ignores the social components in the supply chain network, and SSC was established to address this issue [26]. Seuring and müller [27] were the first to suggest the idea of SSC. Economy, environment, society, and product recycling are the primary research areas of SSC.

As a means of giving businesses a competitive edge, sustainability refers to balancing financial success with social and environmental concerns. Therefore, environmental and social factors must be included in the supplier selection criteria of businesses in addition to the traditional economic reasons [28]. The main goal of supply chain planning is to maximize economic gain [29], and the two most popular optimization indicators are profit [30] and cost [31,32]. Similar to how economic benefits are often considered when making decisions, many researchers also believe in environmental aspects of sustainability. For example, building a tactical supply chain planning model [33] to study the balance between the economy and the environment and using carbon emissions as an indicator of environmental components [34,35] are just two examples. More scholars and practitioners are examining SSC concerns to balance economic, environmental, and social benefits due to consumer attention to environmental and social issues and strengthening government regulation in this area [36].

After selecting appropriate evaluation criteria for suppliers, the distribution of weights is also critical, mainly including subjective and objective weighting methods, which can also be obtained through the combination of these methods [37]. The primary purpose of weight is to distinguish the importance of different criteria according to people’s will. Therefore, subjective weighting is the most commonly used weight setting method [38]. The most widely used method in this category, AHP, is frequently used in supplier selection [15]. It enables the decision-maker to gauge the consistency of their judgments. Additionally, it is user-friendly and appropriate for group choices [39]. On the other hand, subjective approaches rely on individual tastes and struggle with consistency [37]. The objective weighing approach, such as the entropy weight method, is frequently employed to address this issue and make up for the drawbacks of the subjective weight-setting method. The above two methods are sometimes adopted simultaneously for more comprehensive results [40].

Many studies have adopted quantitative methods, focusing on supplier evaluation and selection, and most methods include MCDM, artificial intelligence, and mathematical programming [26]. Govindan pointed out that as a decision-making tool to deal with this problem, MCDM methods are still more popular than mathematical programming and artificial intelligence methods [41]. There will always be some degree of uncertainty in the decision-making process. Because information is sometimes delayed and inconsistent, it can be challenging to determine the criteria [17]. To solve the criteria difficulties, various MCDM approaches have been created. These can be categorized into two groups: integrated MCDM and singular working MCDM methods [42]. Data envelopment analysis (DEA) [2], multi-objective linear programming method [43], analytic hierarchy process (AHP) [44], mathematical programming method [45], the technique for order preference by similarity to ideal solutions (TOPSIS) [46], and decision-making trial and evaluation laboratory (DEMATEL) [47] are examples of single working MCDM methods. Two independent functioning MCDM methods are combined to create integrated MCDM methods [48]. A separate functioning MCDM technique may also be combined with auxiliary algorithms, such as genetic algorithms [49], artificial neural networks [50], cluster analyses [51], and fuzzy theory [52], to form an integrated MCDM method. The integrated MCDM technique can lessen or even eliminate away with the drawbacks of the single MCDM method [42]. Choosing an appropriate MCDM method when solving a multi-attribute decision-making problem is essential. Different MCDM models have their own characteristics, and no MCDM method is superior to the others in all aspects [53].

With the complexity of economic activities, uncertain information increasingly impacts enterprise management. In practice, sustainable supplier selection usually involves decision makers in different departments, such as purchasing, production, quality control departments, etc., and there are many conflicts in the standards of sustainability and risk. Therefore, selecting sustainable suppliers is a frequent and difficult MCDM challenge [54,55]. Several evaluation criteria have altered the evaluation process: the evaluation information’s complexity and the evaluation criteria’ uncertainty [56]. Due to the complexity of actual decision-making situations and the limitations and incomplete data of decision-makers subjective cognition, it is challenging to define decision-makers’ evaluation preferences with precise values [57]. To better deal with such problems, the uncertainty theory has also been utilized [58] in various methods [59]. Regarding the supplier selection process, its advantage is to reduce uncertainty [60] and significantly help decision maker’s deal with inaccurate, ambiguous, and subjective data.

Fuzzy sets and fuzzy logic have since been widely employed to handle uncertainty issues in daily life [61,62]. Scholars have successively optimized fuzzy sets [63,64], such as Fuzzy Sets of Type-2 [65], standard fuzzy sets [66], hesitant fuzzy sets [67], interval-valued intuitionistic fuzzy values [20], and complex Pythagorean fuzzy sets [48]. In conjunction with MCDM techniques like AHP, ANP, DEA, and TOPSIS, fuzzy set theory and its extension have been used to solve supplier selection decision-making problems [68,69]. However the fuzzy set theory is unable to cope with the ambiguous and inconsistent standards of information that exist in reality, and it is also unable to address information discontinuity and consistency. Thus, neutrosophic sets were created by Smarandache [70]. It is a discipline of philosophy, and a mathematical instrument used to investigate the beginning, character, range, and relationship of their various conceptual contents. In contrast to other uncertainty sets, the neutrosophic sets take into account the idea of three parameters and employ three membership degrees to represent ambiguous and inconsistent information. More importantly, there is no relationship between these ideas [71]. Since it is challenging to apply neutrosophic sets to actual engineering and research, neutrosophic sets are expanded to SVNS for the flaws of non-standard subsets of neutrosophic sets. Ye defines the idea of simplified neutrosophic sets and uses an exact number between [0,1] to express its truth value, uncertainty, and fallacy to overcome the problems of inaccurate, uncertain, and inconsistent data [72].

In MCDM tools [73], such as AHP [74], DEMATEL [75], multi-objective optimization based on ratio analysis (MULTIMOORA) [76], and TOPSIS [77,78], methods employing Neutrosophic Sets have been employed. MCDM is a suitable approach to handle supplier selection. One of the most well-known of them is the VIKOR approach (multi-criteria compromise ranking method), which was developed by Opricovic (1998) to address discrete choice problems [79]. It is capable of figuring out a compromise solution to the MCDM issue and maintaining equilibrium between overall happiness and personal regrets [80]. The VIKOR method aids decision-makers in figuring out a compromise solution to the decision-making problem to arrive at a more accurate final decision [39]. It focuses on ranking alternatives and choosing the best from a set of these alternatives, which can include many conflicting and non-commensurable criteria. The VIKOR approach is frequently employed to address issues in management and decision-making [81,82].

Solving sustainability-related problems has become more difficult today due to natural disasters, diseases, climate change, social responsibility, and other variables disrupting the corporate environment. The literature review of suppliers suggests that the idea of sustainability has been independently researched in the context of supplier selection. This research identifies several indicators based on the economic, social, and environmental sustainability elements discussed in the literature. In reality, judgments depend on qualitative data, and decision-makers’ subjective preferences frequently affect how supplier preferences and criteria are weighted [83]. AHP can measure the proportional importance of criteria, and its flexibility and ability to check for inconsistencies can help decision-makers integrate subjectivity, experience, and knowledge into the decision-making process. The entropy weight method is regarded as influential in determining the weight of criteria in an uncertain decision-making environment. Entropy describes the uncertainty of information, increasing the utilization of practical knowledge and ensuring decision-making accuracy.

Therefore, this paper considers the importance of human judgment and the information provided by the original data and uses the AHP and entropy weight methods to obtain the indicators’ total weight. VIKOR method is also increasingly used to quantitatively assess complex economic, social, and environmental processes. In the VIKOR method, the selected best alternative considers the relative closeness to the ideal and harmful solutions and the defined acceptable advantage and acceptable stability in decision-making with three parameters [84]. VIKOR method achieves the optimal ranking of limited alternative decision-making schemes by maximizing the group utility and minimizing the individual regret degree, which makes the decision more reasonable [85]. The MCDM problem’s neutrosophic set approach helps to overcome the drawbacks of integrating qualitative measurement and subjective assessment. To describe the selection problem and do a sensitivity analysis, this paper proposes a VIKOR model based on single-valued neutrosophic set to lessen the impact of individual preference and experience on the final results of evaluation, to solve the issue of excessive personal regret brought on by factor correlation, and put forward the notion of "relative distance" to increase evaluation accuracy. The method proposed in this paper provides a solution closer to ideal and a balance between minimal individual regret and maximum group utility, and also considers the flawed information provided by managers to help decision-makers communicate evaluation information more accurately and effectively. Moreover, this method has lower complexity.

## 3 Basic concept

### 3.1 VIKOR

The core idea of the VIKOR method is to determine the positive and negative solutions, calculate the variables, and sort the schemes. The smaller the value, the better the evaluation object [85]. For the purpose of calculating programs, the following formulas are used:

$$S_{i}=\sum_{j=1}^{n}w_{j}\frac{f_{ij}^{+}-f_{ij}}{f_{ij}^{+}-f_{ij}^{-}}$$

$$R_{i}=\underset{j}{\max}\{w_{j}\frac{f_{ij}^{+}-f_{ij}}{f_{ij}^{+}-f_{ij}^{-}}\}$$

$$Q_{i}=v(\frac{S_{i}-S^{-}}{S^{+}-S^{-}})+(1-v)(\frac{R_{i}-R^{-}}{R^{+}-R^{-}})$$ (1)

Among them, *S*_*i*_ represents the overall utility value and *R*_*i*_ represents the individual regret value. $f_{ij}^{+}$ is the positive rational solution, $f_{ij}^{-}$ is the negative rational solution, and *Q*_*i*_ represents the ratio of benefits that the scheme *A*_*i*_ can produce. $S^{+}=\max S_{i},S^{-}=\min S_{i},R^{+}=\max R_{i},R^{-}=\min R_{i}$. *v* is the adjustment coefficient of “group utility” and “individual regret,” *v*<0.5 means more attention to individual regret and *v*>0.5 means more attention to group satisfaction; *v* usually equals 0.5.

By using linear weighting and only taking into account the difference between each scheme under each index and the optimal term under that index, the conventional VIKOR technique subtracts two values when computing the distance. The acquired solution will raise the calculated value of the group utility, comparable to a covert increase of the group utility coefficient v, leading to a reasonably considerable divergence in the estimated distance. The individual regret value in Formula (1) is to select the most significant value. The classic VIKOR approach will boost the personal regret value and exaggerate the particular regret when the assessment indicators are related.

On the basis of this, the conventional VIKOR approach has to be improved. Three parameters are taken into account in the theory of neutrosophic sets: the degree of truth, the degree of uncertainty, and the degree of distortion. This can assist decision-makers in providing more accurate and thorough opinions as well as addressing issues that the VIKOR is unable to address.

### 3.2 Single-valued neutrosophic sets

**Definition 1.** [76] (SVNS): Let *X* be the given collection, the single-valued neutrosophic *A* is defined in the collection *X*; it consists of three functions $\left(T_{A}(x),I_{A}(x),F_{A}(x)\right)$ of a finite subset of *X* to the unit interval [0,1]. *A* can be expressed as $A=\{\langle x,T_{A}(x),I_{A}(x),F_{A}(x)\rangle x\in X\}$. Here, $T_{A}(x),I_{A}(x),F_{A}(x)$ are three sets of finite discrete values that belong to [0,1], representing the degree of truth, the degree of uncertainty, and the degree of distortion, respectively.

In particular, *a* = 〈*t*,*i*,*f*〉 is called a single-valued neutrosophic number, and $0\leq t,i,f\leq 1,0\leq t^{+}+i^{+}+f^{+}\leq 3,t\in T_{A}(x),i\in I_{A}(x),f\in F_{A}(x)$.

**Definition 2.** [76]: $A=\langle T_{A}(x),I_{A}(x),F_{A}(x)\rangle$ is set to the single-valued neutrosophic set. Hence, the complementary set of *A* is $A^{C}=\langle 1-T_{A}(x),1-I_{A}(x),1-F_{A}(x)\rangle$

**Definition 3.** [86]: For the single-valued neutrosophic number $A=\langle T_{A}(x),I_{A}(x),F_{A}(x)\rangle$, its scorecard function *S*_*A*_, exact function *α*_*A*_, and definite function *C*_*A*_ are respectively defined as follows:

$$S_{A}=\frac{T_{A}+1-I_{A}+1-F_{A}}{3}$$

$$\alpha_{A}=T_{A}-F_{A}$$

$$C_{A}=T_{A}$$ (2)

**Definition 4.** [86]: For two single-valued neutrosophic numbers *A* and *B*:

If *S*_*A*_>*S*_*B*_, then *A*>*B*;

If $S_{A}=S_{B},\alpha_{A}>\alpha_{B}$, then *A*>*B*;

If $S_{A}=S_{B},\alpha_{A}=\alpha_{B},C_{A}>C_{B}$, then *A*>*B*;

The following formula is used to calculate the distance between single-valued neutrosophic sets:

$$A=\{T_{A}(x),I_{A}(x),F_{A}(x)|x_{i}\in X)\},B=\{T_{B}(x),I_{B}(x),F_{B}(x)|x_{i}\in X)\},$$

Then, the weighted measurement distance formula between the two single-valued neutrosophic set is expressed as [87]:

$$d_{\lambda }(A,B)={\left[\sum_{j=1}^{n}W_{j}{\left(\sum_{i=1}^{4}\beta_{i}\phi_{i}(x_{j})\right)}^{\lambda }\right]}^{\frac{1}{\lambda }}$$ (3)

Among, $\lambda >0,\beta_{i}\in [0,1],\sum_{i=1}^{4}\beta_{i}=1,W_{j}\in [0,1]$ and $\sum_{j=1}^{n}W_{j}=1$,

$$\phi_{1}(x_{j})=\frac{\left|T_{A}(x_{i})-T_{B}(x_{i})\right|}{3}+\frac{\left|I_{A}(x_{i})-I_{B}(x_{i})\right|}{3}+\frac{\left|F_{A}(x_{i})-F_{B}(x_{i})\right|}{3}$$

$$\phi_{2}(x_{j})=\max\{\frac{2+T_{A}(x_{i})-I_{A}(x_{i})-F_{A}(x_{i})}{3},\frac{2+T_{B}(x_{i})-I_{B}(x_{i})-F_{B}(x_{i})}{3}\}$$

$$-\min\{\frac{2+T_{A}(x_{i})-I_{A}(x_{i})-F_{A}(x_{i})}{3},\frac{2+T_{B}(x_{i})-I_{B}(x_{i})-F_{B}(x_{i})}{3}\}$$

$$\phi_{3}(x_{j})=\frac{\left|T_{A}(x_{i})-T_{B}(x_{i})+I_{B}(x_{i})-I_{A}(x_{i})\right|}{2}$$

$$\phi_{4}(x_{j})=\frac{\left|T_{A}(x_{i})-T_{B}(x_{i})+F_{B}(x_{i})-F_{A}(x_{i})\right|}{2}$$

## 4 Model construction

Since numerous indicators and evaluation topics are involved in evaluating sustainable suppliers, it is vital to weigh both precise and ambiguous information concurrently [88]. This leads to establishing the three-phased proposed model for sustainable supplier selection in an uncertain environment:

- Building the sustainable supplier evaluation index system
- Obtaining the sustainable supplier evaluation index criteria weights
- Ranking potential sustainable suppliers

### 4.1 Construction of index system

In the conventional assessment procedure, we must first choose the best evaluation criteria before evaluating the alternatives and selecting the supplier. The creation of appropriate measures is a significant obstacle in supplier selection. These criteria are often seen to include three areas: the economy (money supply and capital), society (social issues), and the environment (pollution).As a result, sustainable providers should consider sustainability of the economy, society, and environment. Economic sustainability aims to maximize profits with the least amount of capital invested [89]; environmental sustainability concentrates on environmental issues [56] like pollution, resource reuse, and resource consumption; it also pays attention to resource utilization efficiency and waste generation; and social sustainability concentrates on employee equity and social responsibility. To produce unbiased and fair evaluation results, the sustainable supplier evaluation method must adhere to rational and scientific standards. The index system is made in accordance with the principles of hierarchy and systematization, independence, authenticity, and comparability, as well as the combination of qualitative and quantitative principles, and the evaluation system is built in accordance with practice. See Table 1.

**Table 1:** Sustainable supplier evaluation index system.

Dimension	Index layer	Detailed indicators
Economy *B*_*1*_	Cost [13,90–95] *C*_*1*_	Product Price [12,56,92,96–101], Product costs [74,101–104], Ordering Costs [74,101–104], Transportation Costs [74,88,97–101,103–107] Carbon tax [96]
	Innovation Capability *C*_*2*_	Innovativeness [13,74,93,101,108,109], Technical Capabilities [12,74,90,91,94,95,97,99,110,111], Patents [102]
	Quality [13,32,56,90–95,97] *C*_*3*_	Product Qualification Rate [94,98,102,112], Product Safety [56,100], Product Quality Improvement [92,98,113], Defect rate [74,103,112], Rejection rate [97,99,101,114], Quality Management System [98,100,101,104,115–117]
	Service Capability [91,94,98,118] *C*_*4*_	Delivery Period [32,56,97,106,109], On-time delivery [74,86,90,92,97,100,103,107,109,112,113,116,117,119], After-sales service level [32,104,106,116,117,119], Order Completion Rate [103], Flexibility [12,56,86,90–92,96,110,114,115], Logistics [107,116], Responsiveness [99,104,107,119]
	Long-term cooperation [116] *C*_*5*_	Finance [90,94–96,100,104,109,110,112,115,116], Culture [90], Enterprise size [102], Relationships [90,99,100,109,110,115,118], Performance History [86], Credibility [74,100], Supply capacity [92,117,118], Production facilities [74,94,100,109,112,116], Risks [32,90,104,112], Public Relations Capability
Society *B*_*2*_	Environmental Management System [92,97,107,115] *C*_*6*_	Environmental Management System [74,89–91,94,96,99,100,110,114,118,120], Eco-design [13,56,74,95,97,99,102,114,120], environmental certification [74,99,100,102,109], environmental costs [89,110], employee environmental training [98,100,109,120]
	Reduce pollution [99] *C*_*7*_	Greenhouse gas emissions [90,109,121], Pollution Control [13,89,90,94,96,97,100,107,109–111,114,115,118], Resource consumption [12,74,90,91,98–100,103,107,108,110,111,115], Green Logistics [96,113], Reuse/recycling [74,90,91,99,107,109], Harmful substances [56,98,107,109,115], Waste Management [97–99,104,107,110]
	Green Image [90,92,94,98,100,114,120] *C*_*8*_	Green Production [98,104,113,120], Green Packaging [74,99,100,104,110,114], Environmentally friendly materials [74,92,99,120], Green Products [94,96,97,111,114,115], Green Technology Capability [96,99,100,107–110,115],
Environment *B*_*3*_	Social Responsibility [56,90,94,96,100,121] *C*_*9*_	Impact of local establishment [74,90,94,97,110,115,118,122], Social Welfare [12,90,98], Employment Opportunities [90,99,102], Ethical issues and legal compliance [56,74,100,107,109,110,114,123], Product Liability [93,109],
	Employment Practices [93,122] *C*_*10*_	Employee Rights and Benefits [74,89–92,99,104,110,111,113,115,121,122], Work safety and labor health [13,74,89,90,92–97,99–102,105–107,113–115,118,121], Working Environment (Human rights, discrimination, forced labor) [93,98,99,106,109,113,118], Employee turnover rate [74,98], Stakeholder Power [74,89–91,94,98,99,102,104,107,109–111,114,115,118,122], Information Disclosure [91,96,98,104,107,110,114,122], Staff Training [89,95,97,99,104,107,110,115,118]

### 4.2 Comprehensive weight method

#### 4.2.1 Calculating subjective weight by AHP

AHP is a weighing technique that has quickly evolved for usage in real-world applications [124]. Following are the steps for utilizing the AHP approach to determine the weight of the criteria:

**Step 1.** Create a decision hierarchy using the requirements and options.
**Step 2.** Create the matrix for comparisons. As paired comparisons, each standard is evaluated versus various other criteria. All comparison matrices should be finished in accordance with the Saaty scale (Table 2), as follows [125]:

$$A=(\begin{array}{cccc} 1 & \frac{c_{1}}{c_{2}} & \cdots & \frac{c_{1}}{c_{n}}\\ \frac{c_{2}}{c_{1}} & 1 & \cdots & \frac{c_{2}}{c_{n}}\\ \vdots & \vdots & 1 & \vdots \\ \frac{c_{n}}{c_{1}} & \frac{c_{n}}{c_{2}} & \cdots & 1 \end{array})$$

**Table 2:** Scale given by reference [126] for pairwise comparison.

Preference/ordinal scale	Intensity of importance
1	Equal
3	Moderate
5	Strong
7	Very strong
9	Extreme
2,4,6,8	Intermediate weights between 1.3.5.7,9
Reciprocals	Inverse (opposite) comparison

**Step 3.** Different weights can be applied to the sub-criteria and factors based on the various input data, and the correct weights can be derived. The results of the criterion were determined by creating a cumulative pairwise comparison matrix using the AHP software.
**Step 4.** Check the comparability matrices for consistency. The consistency ratio (CR), which is determined by dividing the consistency index (CI) by the random index (RI), determines the surface of the comparison matrix [125]. RI is influenced by matrix order (n). RI is calculated using Table 3. Where n stands for the number of criteria and is the matrix’s primary eigenvalue. AHP software’s reported incompatibility rate is assessed. A valid conclusion can be made if the inconsistency rate is 0.1 or less and the matrix is consistent.

**Table 3:** Presents a random index from reference [125].

n	2	3	4	5	6	7	8	9	10
RI	0	0.52	0.89	1.11	1.25	1.35	1.40	1.45	1.49

#### 4.2.2 Calculating objective weight by entropy

Let the neutrosophic sets be $A=\{x,T_{A}(x),I_{A}(x),F_{A}(x)|x_{i}\in X\}$ and the domain be *X* = {*x*_1_,*x*_2_,⋯*x*_*m*_}, then calculate the entropy *E*_(*A*)_ of each single-valued neutrosophic set in the evaluation matrix. Determine the factor weight as Eq (4) [126].


$$E_{\left(A\right)}=1-\frac{1}{n}\sum_{x_{i}\in x}\left(T_{A}(x_{i})+F_{A}(x_{i})\cdot |I_{A}(x_{i})-I_{A^{C}}(x_{i})|\right)$$

$$o\omega_{c_{j}}=\frac{1-\frac{1}{m}\sum_{i=1}^{m}E_{\left(A\right)}}{\sum_{j=1}^{n}\left(1-\frac{1}{m}\sum_{i=1}^{m}E_{\left(A\right)}\right)}(i=1,2,\cdots ,m;j=1,2,\cdots n)$$ (4)

Among, *E*_(*A*)_ is the entropy of the neutrosophic set *A*, $o\omega_{c_{j}}$ is the subjective weight of the index.

#### 4.2.3 Obtaining the combination weight

The comprehensive weight of indicators (*c*_*j*_) can be expressed by Eq in order to increase judgment accuracy and get beyond the limitations of the independent subjective weighting approach and the independent objective weighting method. (5) [38,127].


$$\omega_{c_{j}}=\alpha \times s\omega_{c_{j}}(1-\alpha )\times o\omega_{c_{j}}\left(j=1,2,\cdots ,n\right)$$ (5)

Whereas $\omega_{c_{j}}$ is the comprehensive weight, $o\omega_{c_{j}}$ is the objective weight, $s\omega_{c_{j}}$ is the subjective weight obtained by the AHP method, and α is weight coefficient, 0≤*α*≤1. Here we take *α* = 0.5.

### 4.3 VIKOR method based on single valued neutrosophic set

One must consider both the distance between A and B and the spread between A and B and C while extending the single-valued neutrosophic sets to VIKOR. To ensure that the distance between each scheme and the positive ideal solution is minimized and the distance between each project and the perfect negative solution is always the greatest, the weighted relative distance between each answer and the positive and negative ideal solutions is divided by the weighted distance between the positive and negative perfect solutions to determine the "relative distance."

The following formulas are used:

$$\Delta d_{ij}=d_{ij}^{+}-d_{ij}^{-}$$

$$d_{ij}^{+}=d(O_{ij},O_{ij}^{+})=d(O_{ij}^{+},O_{ij})$$

$$d_{ij}^{-}=d(O_{ij},O_{ij}^{-})=d(O_{ij}^{-},O_{ij})$$ (6)

Here, Δ*d*_*ij*_ is a relative distance, and the smaller Δ*d*_*ij*_ is, the better the scheme is. On the contrary, the larger Δ*d*_*ij*_ is, the worse the scheme is, *i* = 1,2,⋯,*m*, *j* = 1,2,⋯,*n*.

In a single valued neutrosophic sets environment, this study assumes that there are *m* alternative schemes, and that the alternative set is $A=\{A_{1},A_{2}\cdots ,A_{n}\}$, and *n* indicators are selected to evaluate each scheme. The index set is $C=\{C_{1},C_{2}\cdots ,C_{n}\}$, and the *n* attribute values of scheme *A*_*i*_ are represented by $O=\{O_{i1},O_{i2},\cdots ,O_{in}\}$, where *O*_*ij*_ is the value of the scheme *A*_*i*_ under the *C*_*j*_ index, expressed in the form of the a single valued neutrosophic number, that is:

$O_{ij}=\langle T_{A_{i}}(c_{j}),I_{A_{i}}(c_{j}),F_{A_{i}}(c_{j})\rangle$, where *i* = 1,2,⋯,*m*, *j* = 1,2,⋯,*n*, the weight of each index is determined using the entropy weight method.

According to definitions 3 and 4, the positive ideal solution $O_{ij}^{+}$ and negative ideal solution $O_{ij}^{-}$ of *m* alternatives are determined, and the weighted distance $\Delta d_{ij}^{*}$ between them is calculated by Formula (3), *i* = 1,2,⋯,*m*, *j* = 1,2,⋯,*n* .

Using Formula (3) to calculate the weighted distance $d_{ij}^{+}$ of each scheme and positive ideal solution $O_{ij}^{+}$, *i* = 1,2,⋯,*m*, *j* = 1,2,⋯,*n*.

Using Formula (3) to calculate the weighted distance $d_{ij}^{-}$ of each scheme and negative scenario solution $O_{ij}^{-}$, *i* = 1,2,⋯,*m*, *j* = 1,2,⋯,*n*.

Δ*d*_*ij*_ is calculated according to Formula (6).

According to the formula, *S*_*i*_, *R*_*i*_ and *Q*_*i*_ are calculated. The decision-making mechanism coefficient *v* in the formula takes the median value, order *v* = 0.5, which means that the maximum group utility should be pursued and the individual regret should be minimized at the same time.

The calculation formulas of *S*_*i*_, *R*_*i*_ and *Q*_*i*_ of the scheme are as follows:

$$S_{i}=\sum_{j=1}^{n}W_{j}\frac{\Delta d_{ij}}{\Delta d_{ij}^{*}}$$

$$R_{i}=\underset{j}{\max}\langle W_{j}\frac{\Delta d_{ij}}{\Delta d_{ij}^{*}}\rangle$$

$$Q_{i}=v(\frac{S_{i}-S^{-}}{S^{+}-S^{-}})+(1-v)(\frac{R_{i}-R^{-}}{R^{+}-R^{-}})$$ (7)

Among them, *S*_*i*_ represents the overall utility value, and *R*_*i*_ represents the individual regret value. Δ*d*_*ij*_ represents the relative distance between each scheme and the positive and negative ideal solutions, $\Delta d_{ij}^{*}=d(O_{ij}^{+},O_{ij}^{-})$ represents the relative distance between positive and negative ideal solutions, and *Q*_*i*_ represents the benefit ratio that can be generated by scheme *A*_*i*_,

*S*^+^ = max *S*_*i*_, *S*^−^ = min *S*_*i*_, *R*^+^ = max *R*_*i*_, *R*^−^ = min *R*_*i*_, *i* = 1,2,⋯,*m*, *j* = 1,2,⋯,*n*, *v* denotes the decision-making mechanism coefficient, which is equivalent to a weight. When *v*∈[0,0.5], it is more focused on the pursuit of the group effect; when *v*∈[0.5,1], it means more attention should be paid to individual regret.

The compromise solution in the VIKOR approach is established based on two decision conditions.

Condition 1: Describe it as an acceptable minimal requirement, i.e.


$$Q^{2}-Q^{1}\geq DQ$$

$$DQ=\frac{1}{M-1}$$ (8)

Ranking from small to large according to the *Q*_*i*_, symbol description in Formula (8) is that:

*Q*^1^: the *Q* value of scheme *A*_1_ which is ranked first,
*Q*^2^: the *Q* value of scheme *A*_2_ which is ranked after *A*_1_,
*M*: The total number of options available.

Only when $Q^{2}-Q^{1}\geq \frac{1}{M-1}$ can it be said that option *A*_1_, which ranks first, has an obvious advantage over alternative option *A*_2_.

If there is more than one sort result, *A*_1_ need to be changed with *A*_2_,*A*_3_,⋯,*A*_*n*_, one-to-one calculations should be done to determine if they meet the first condition.

Condition 2: It is called the acceptable decision reliability in the decision process.

After sorting by *Q* value, $S_{A_{1}}$ must be less than $S_{A_{2}}$ at the same time, or $R_{A_{1}}$ must also be less than $R_{A_{2}}$.

As with condition 1, if more than one result occurs, *A*_1_ need to be changed with *A*_2_,*A*_3_,⋯,*A*_*n*_, one by one to see if they meet the second condition.

The VIKOR compromise solution is shown in Table 4.

**Table 4:** VIKOR compromise solution.

Judgment Criterion		Result	Optimal Compromise Solution
The relationship between *A*_1_ and *A*_2_schemes satisfies both condition 1 and condition 2.		To determine that scheme *A*_1_ is a reliable and optimal scheme to choose from.	*A*_1_
The value of the relationship Between *A*_1_ and *A*_2_ satisfies one of the conditions	It only satisfies condition 1, it does not satisfy condition 2	At the same time, both schemes *A*_1_ and *A*_2_ are found to be optimal.	*A*_1_ or *A*_2_
	It only satisfies condition 2, it does not satisfy condition 1	At the same time, identify those schemes *A*_2_,*A*_3_,⋯,*A*_*J*_ that do not meet condition 1, is the best scheme.	*A*_2_,*A*_3_,⋯,*A*_*J*_

Note: *A*_1_ and *A*_2_ are the alternatives of the first and second positions after the C values are sorted from small to large; *A*_*J*_ is the maximum *J* value determined by *Q*^*j*^−*Q*^1^<*DQ*.

### 4.4 Supplier evaluation system

**Fig 1:**
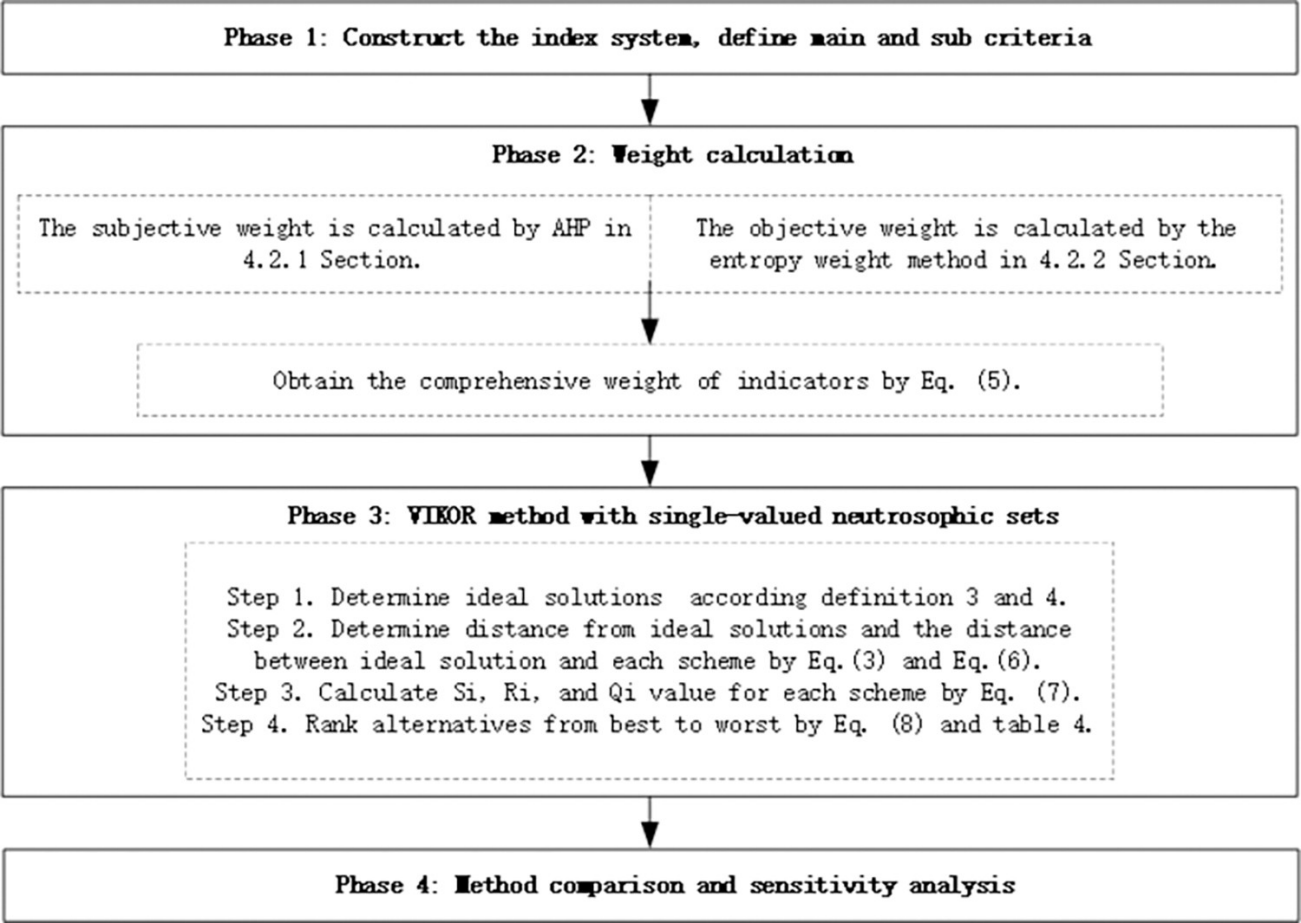
The proposed methodology.

Fig 1 presents the suggested integrated methodology, and it is theoretically explained in the steps that follow.

## 5 Numerical study

Due to the need for strategic development, an enterprise *K* determines that strengthening sustainable supply chain management requires a long-term, stable sustainable supplier. After enterprise inspection, there are four alternative suppliers *A*_1_, *A*_2_, *A*_3_, and *A*_4_ to meet the requirements of the enterprise. It is necessary to evaluate these to select those that fit the long-term development of enterprise *K*.

### 5.1. The weight calculation

Step 1. Calculating the subjective weight of the indications utilizing the methods in Section 4.2.1. To obtain the final weight of the index, the pairwise comparison matrix utilized the conventional Saaty’s scale (from Table 2). The results are shown in Tables 5–8.

**Table 5:** Comparison matrix and weights of criteria *B*_1_.

Criteria	*c*_1_	*c*_2_	*c*_3_	*c*_4_	*c*_5_	Weights	CR
*c*_1_	1	2	2	3	3	0.3614	0.0927435
*c*_2_	1/2	1	1/3	1/3	3	0.1233	
*c*_3_	1/2	3	1	2	2	0.2526	
*c*_4_	1/3	3	1/2	1	2	0.1765	
*c*_5_	1/3	1/3	1/2	1/2	1	0.0862	

**Table 6:** Comparison matrix and weights of criteria *B*_2_.

Criteria	*c*_6_	*c*_7_	*c*_8_	Weights	CR
*c*_6_	1	1/3	2	0.2493	0.0515592
*c*_7_	3	1	3	0.59336	
*c*_8_	1/2	1/3	1	0.1571	

**Table 7:** Comparison matrix and weights of criteria *B*_3_.

Criteria	*c*_9_	*c*_10_	Weights	CR
*c*_9_	1	1/3	0.25	0
*c*_10_	3	1	0.75	

**Table 8:** Comparison matrix and weights of criteria dimension.

Criteria	*B*_1_	*B*_2_	*B*_3_	Weights	CR
*B*_1_	1	2	4	0.5584	0.0175911
*B*_2_	1/2	1	3	0.3196	
*B*_3_	1/4	1/3	1	0.122	

The final subjective weight of each indicator is: $s\omega_{c_{1}}=0.2018$, $s\omega_{c_{2}}=0.0689$, $s\omega_{c_{3}}=0.141$, $s\omega_{c_{4}}=0.0986$, $s\omega_{c_{5}}=0.0481$, $s\omega_{c_{6}}=0.0797$, $s\omega_{c_{7}}=0.1897$, $s\omega_{c_{8}}=0.0502$, $s\omega_{c_{9}}=0.0305$, $s\omega_{c_{10}}=0.0915$.

Step 2. Calculating the objective weight of indicators. For sustainable suppliers, *A*_1_, *A*_2_, *A*_3_, or *A*_4_ are chosen, the candidate suppliers are evaluated and selected using the 10 indicators above, *c*_1_~*c*_10_. Indicator values of each scheme $A_{i}$ under each index $c_{j}$ are expressed as single valued neutrosophic sets, $O_{ij}=\langle T_{A_{i}}(c_{j}),I_{A_{i}}(c_{j}),F_{A_{i}}(c_{j})\rangle$. Among them, *i* = 1,2,⋯,*m*, *j* = 1,2,⋯,*n*, the data obtained are reported in Table 9.

**Table 9:** Data for suppliers under each index in a SVNS environment.

Plan Index	*A*_1_	*A*_2_	*A*_3_	*A*_4_
*c*_1_	<0.9,0.1,0.2>	<0.8,0.1,0.2>	<0.8,0.1,0.1>	<0.8,0.1,0.2>
*c*_2_	<0.9,0.1,0.1>	<0.9,0.2,0.2>	<0.8,0.2,0.2>	<0.7,0.1,0.1>
*c*_3_	<0.8,0.2,0.3>	<0.8,0.1,0.3>	<0.7,0.2,0.3>	<0.8,0.2,0.3>
*c*_4_	<0.7,0.2,0.4>	<0.7,0.2,0.3>	<0.6,0.3,0.4>	<0,7,0.2,0.4>
*c*_5_	<0.5,0.4,0.4>	<0.6,0.4,0.5>	<0.5,0.3,0.4>	<0.6,0.3,0.4>
*c*_6_	<0.7,0.2,0.1>	<0.8,0.2,0.2>	<0.8,0.2,0.3>	<0.8,0.2,0.2>
*c*_7_	<0.8,0.1,0.2>	<0.8,0.1,0.1>	<0.9,0.1,0.3>	<0.9,0.2,0.3>
*c*_8_	<0.5,0.4,0.4>	<0.6,0.3,0.4>	<0.5,0.4,0.3>	<0.5,0.4,0.5>
*c*_9_	<0.4,0.3,0.3>	<0.5,0.3,0.4>	<0.6,0.3,0.4>	<0.5,0.3,0.5>
*c*_10_	<0.6,0.3,0.4>	<0.7,0.4,0.4>	<0.5,0.2,0.3>	<0.6,0.3,0.4>

The objective weight of each indicator is determined according to Formula (4), and the objective weight of each indicator is: $o\omega_{c_{1}}=0.1481$, $o\omega_{c_{2}}=0.125$, $o\omega_{c_{3}}=0.1296$, $o\omega_{c_{4}}=0.1074$, $o\omega_{c_{5}}=0.0537$, $o\omega_{c_{6}}=0.1083$, $o\omega_{c_{7}}=0.1481$, $o\omega_{c_{8}}=0.0435$, $o\omega_{c_{9}}=0.0667$, $o\omega_{c_{10}}=0.0694$.

Step 3. Obtaining the combination weight by Eq (5), and the combination weight of each indicator is: $\omega_{c_{1}}=0.175$, $\omega_{c_{2}}=0.097$, $\omega_{c_{3}}=0.1353$, $\omega_{c_{4}}=0.103$, $\omega_{c_{5}}=0.0509$, $\omega_{c_{6}}=0.094$, $\omega_{c_{7}}=0.1689$, $\omega_{c_{8}}=0.0469$, $\omega_{c_{9}}=0.0486$, $\omega_{c_{10}}=0.0805$.

### 5.2. Evaluation and Selection Method of VIKOR with SVNS

The positive ideal solution $O_{ij}^{+}$ and the negative ideal solution $O_{ij}^{-}$ of the four sustainable suppliers are determined according to definitions 3 and 4, respectively.


$$O_{ij}^{+}=\langle \begin{array}{c} \left(0.9,0.1,0.2),(0.9,0.1,0.1),(0.8,0.1,0.3),(0.7,0.2,0.3),(0.6,0.3,0.4\right)\\ \left(0.8,0.2,0.2),(0.8,0.1,0.1),(0.6,0.3,0.4),(0.6,0.3,0.4),(0.5,0.2,0.3\right) \end{array}\rangle$$

$$O_{ij}^{-}=\langle \begin{array}{c} \left(0.8,0.1,0.2),(0.8,0.2,0.2),(0.7,0.2,0.3),(0.6,0.3,0.4),(0.5,0.4,0.4\right)\\ \left(0.8,0.2,0.3),(0.9,0.2,0.3),(0.5,0.4,0.5),(0.5,0.3,0.5),(0.6,0.3,0.4\right) \end{array}\rangle$$

The weighted distance of positive and negative ideal solutions under each index is calculated according to Formula (3), in which the weight of each index $w_{c_{i}}$ has been calculated using the entropy weight method. At this point, λ=2,β=14, and the distance are then obtained as follow.

$\Delta d_{1}^{*}=0.017429$, $\Delta d_{2}^{*}=0.031137$, $\Delta d_{3}^{*}=0.026056$, $\Delta d_{4}^{*}=0.032094$, $\Delta d_{5}^{*}=0.015981$

$\Delta d_{6}^{*}=0.08943$, $\Delta d_{7}^{*}=0.025688$, $\Delta d_{8}^{*}=0.021647$, $\Delta d_{9}^{*}=0.015613$, $\Delta d_{10}^{*}=0.09456$

The comprehensive distance between positive and negative ideal solutions is $d_{\lambda }(O_{ij}^{+},O_{ij}^{-})=0.069132$ The same method determines $d_{ij}^{+}$ and $d_{ij}^{-}$. The results are shown in Tables 10 and 11.

**Table 10:** The weighted distance between each scheme and the ideal solution under each index $d_{ij}^{+}$.

Plan Index	*A*_1_	*A*_2_	*A*_3_	*A*_4_
*c*_1_	0.000000	0.017429	0.012200	0.017429
*c*_2_	0.000000	0.018163	0.031137	0.025947
*c*_3_	0.010729	0.000000	0.026056	0.010729
*c*_4_	0.009361	0.000000	0.032094	0.009361
*c*_5_	0.015981	0.013161	0.009401	0.000000
*c*_6_	0.008943	0.000000	0.008943	0.000000
*c*_7_	0.011988	0.000000	0.023975	0.025688
*c*_8_	0.015333	0.000000	0.012627	0.021647
*c*_9_	0.015613	0.009184	0.000000	0.015613
*c*_10_	0.009456	0.017730	0.000000	0.009456
Total	0.035356682	0.03472135	0.061073098	0.051394557

**Table 11:** The weighted distance between each scheme and the ideal solution under each index $d_{ij}^{-}$.

Plan Index	*A*_1_	*A*_2_	*A*_3_	*A*_4_
*c*_1_	0.017429	0.000000	0.012200	0.000000
*c*_2_	0.031137	0.012974	0.000000	0.010379
*c*_3_	0.015327	0.026056	0.000000	0.015327
*c*_4_	0.022733	0.032094	0.000000	0.022733
*c*_5_	0.000000	0.006580	0.006580	0.015981
*c*_6_	0.017886	0.008943	0.000000	0.008943
*c*_7_	0.013700	0.025688	0.011988	0.000000
*c*_8_	0.006314	0.021647	0.012627	0.000000
*c*_9_	0.012858	0.006429	0.015613	0.000000
*c*_10_	0.000000	0.008274	0.009456	0.000000
Total	0.052323945	0.056910428	0.02878332	0.034566212

On the basis of Tables 10 and 11, Formula (6) is used to calculate Δ*d*_*ij*_, and the results are reported in Table 12.

**Table 12:** Relative weighted distance between each scheme and positive and negative ideal solutions.

Plan Index	*A*_1_	*A*_2_	*A*_3_	*A*_4_
*c*_1_	-0.017429	0.017429	0.000000	0.017429
*c*_2_	-0.031137	0.005189	0.031137	0.015568
*c*_3_	-0.004598	-0.026056	0.026056	-0.004598
*c*_4_	-0.013373	-0.032094	0.032094	-0.013373
*c*_5_	0.015981	0.006580	0.002820	-0.015981
*c*_6_	-0.008943	-0.008943	0.008943	-0.008943
*c*_7_	-0.001713	-0.025688	0.011988	0.025688
*c*_8_	0.009020	-0.021647	0.000000	0.021647
*c*_9_	0.002755	0.002755	-0.015613	0.015613
*c*_10_	0.009456	0.009456	-0.009456	0.009456

According to Formula (7), *S*_*i*_, *R*_*i*_ and *Q*_*i*_, the decision-making coefficient is calculated, that is, *v* = 0.5, the mean value of the decision-making coefficient *v* in used is to maximize the utility of the group and minimize the regret of the individual. The results are reported in Table 13.

**Table 13:** Results of VIKOR method in a SVNS environment.

Plan	*S*_*i*_	*R*_*i*_	*Q*_*i*_
*A*_1_	-0.284167	0.080601	0
*A*_2_	-0.24627	0.175249	0.528218
*A*_3_	0.387335	0.135556	0.790309
*A*_4_	0.359005	0.175249	0.978905
**Rank**	*A*_1_>*A*_2_>*A*_4_>*A*_3_	*A*_1_>*A*_3_>*A*_2_>*A*_3_	*A*_1_>*A*_2_>*A*_3_>*A*_4_

According to the results of Table 13, the compromise solution and the optimal scheme are determined. From Table 13, it can be concluded that scheme *A*_1_ ranks first and scheme *A*_2_ ranks second. Next, it is necessary to verify whether conditions 1 and 2 are met. Calculated by Formula (8): $Q^{2}-Q^{1}=0.528218>DQ=\frac{1}{3}$, condition 1 is satisfied. At the same, *S*_1_<*S*_2_ and *R*_1_<*R*_2_ can be seen from *S*_*i*_ and *R*_*i*_ values at the same time, so it also satisfies condition 2. That is, the top-ranked option *A*_1_ satisfies both requirements of the VIKOR judgment simultaneously and is, therefore, the best sustainable provider to select following the evaluation and ranking of the VIKOR evaluation model in the context of single-valued neutrosophic sets.

### 5.3 Sensitivity analysis

In the actual selection process of the sustainable supplier, affected by the external environment, the company’s decision-makers constantly adjust their decision-making intention, and parameter adjustment will lead to changes in the ranking of alternatives. Therefore, the decision result must have certain robustness. There are two critical parameters; the first is the coefficient of combination weighting of Eq (5), this parameter determines the proportion of emotional weight and objective weight; the second is the coefficient of Eq (7), this parameter determines the balance of the group satisfaction and individual regret.

This paper utilized set α=(0,0.1,0.2,0.3,0.4,0.5,0.6,0.7,0.8,0.9,1) respectively, then analyze how weight adjustments can impact the rankings on our model, which can be seen in Table 14. From the Table 14, alternative suppliers *A*_1_ is always the best. As a result, the ideal outcome is quite stable.

**Table 14:** The compromise measures calculated by *α* at different values.

*α*	0	0.1	0.2	0.3	0.4	0.5	0.6	0.7	0.8	0.9	1
$Q_{A_{1}}$	0.0000	0.0000	0.0000	0.0000	0.0000	0.0000	0.0000	0.0000	0.0000	0.0000	0.0000
$Q_{A_{2}}$	0.5287	0.5285	0.5284	0.5282	0.5281	0.5279	0.5277	0.5276	0.5269	0.5261	0.5254
$Q_{A_{3}}$	0.8824	0.8611	0.8414	0.8231	0.8061	0.7902	0.7753	0.7614	0.7391	0.7159	0.6935
$Q_{A_{4}}$	0.9276	0.9369	0.9464	0.9562	0.9663	0.9766	0.9872	0.9982	1.0000	1.0000	1.0000
$S_{A_{1}}$	-0.3059	-0.3017	-0.2974	-0.2931	-0.2888	-0.2845	-0.2802	-0.2760	-0.2717	-0.2674	-0.2631
$S_{A_{2}}$	-0.2647	-0.2547	-0.2247	-0.1747	-0.1047	-0.0147	0.0953	0.2253	0.3753	0.5453	0.7353
$S_{A_{3}}$	0.4129	0.4079	0.4029	0.3980	0.3930	0.3880	0.3831	0.3781	0.3731	0.3682	0.3632
$S_{A_{4}}$	0.3088	0.3183	0.3279	0.3374	0.3470	0.3566	0.3661	0.3757	0.3853	0.3948	0.4044
$R_{A_{1}}$	0.0694	0.0717	0.0739	0.0761	0.0783	0.0805	0.0827	0.0849	0.0871	0.0893	0.0915
$R_{A_{2}}$	0.1481	0.1581	0.1881	0.2381	0.3081	0.3981	0.5081	0.6381	0.7881	0.9581	1.1481
$R_{A_{3}}$	0.1296	0.1308	0.1319	0.1330	0.1342	0.1353	0.1365	0.1376	0.1387	0.1399	0.1410
$R_{A_{4}}$	0.1481	0.1535	0.1589	0.1642	0.1696	0.1750	0.1803	0.1857	0.1911	0.1964	0.2018

Next, this paper initially set v=(0,0.1,0.2,0.3,0.4,0.5,0.6,0.7,0.8,0.9,1) respectively, then analyze how weight adjustments can directly affect the rankings on our model, which can be seen in Table 15. From Table 15, alternative suppliers *A*_1_ and *A*_2_ are the best when *v* takes values of 0.8 and 0.9, and alternative suppliers *A*_1_ is the best when *v* takes other values, but the best option is always alternative suppliers *A*_1_. Therefore, the optimal result is relatively stable.

**Table 15:** The compromise measures calculated by *v* at different values.

*v*	0	0.1	0.2	0.3	0.4	0.5	0.6	0.7	0.8	0.9	1
$Q_{A_{1}}$	0.0000	0.0000	0.0000	0.0000	0.0000	0.0000	0.0000	0.0000	0.0000	0.0000	0.0000
$Q_{A_{2}}$	1.0000	0.9056	0.8112	0.7167	0.6223	0.5279	0.4335	0.3391	0.2447	0.1502	0.0558
$Q_{A_{3}}$	0.5803	0.6223	0.6643	0.7062	0.7482	0.7902	0.8321	0.8741	0.9161	0.9580	1.0000
$Q_{A_{4}}$	1.0000	0.9953	0.9906	0.9860	0.9813	0.9766	0.9719	0.9672	0.9626	0.9579	0.9532

In conclusion, the ideal result is quite consistent. The decision model suggested in this work can make sure that the decisions made have strong robustness, and alternative providers are the best sustainable suppliers *A*_1_.

### 5.4 Comparison with other methods

The following strategies are utilized for comparative analysis as there are numerous ways to research this area [128–132].

#### 5.4.1 Comparison between the improved and traditional VIKOR methods

The results of the traditional VIKOR approach [128] are presented in Table 16.

**Table 16:** Results and type of traditional VIKOR method.

Plan	*S*_*i*_	*R*_*i*_	*Q*_*i*_
*A*_1_	0.470796583	0.092823978	0.027880
*A*_2_	0.453336601	0.175249442	0.500000
*A*_3_	0.766468911	0.15791948	0.894875
*A*_4_	0.687600923	0.175249442	0.874066
**Rank**	*A*_2_>*A*_1_>*A*_4_>*A*_3_	*A*_1_>*A*_3_>*A*_2_>*A*_4_	*A*_1_>*A*_2_>*A*_4_>*A*_3_

From Table 16, it can be seen that scheme *A*_1_ ranks first and scheme *A*_2_ ranks second. Next, whether the two schemes satisfy two conditions needs to be verified. Calculated by Formula (8): $Q^{2}-Q^{1}=0.4721>DQ=\frac{1}{3}$; condition 1 is satisfied. However *S*_1_>*S*_2_, so condition 2 is not satisfied. Therefore, the traditional VIKOR method is used to sort the evaluation, and the compromise solution is as follows: *A*_1_ and *A*_2_.

In conclusion, although though the findings of the improved VIKOR method are essentially the same as those of the conventional VIKOR method, there is only one scheme that is the improved VIKOR method’s ideal solution, and that is *A*_1_.

Although the compromise solutions *A*_1_ and *A*_2_ are obtained using the traditional VIKOR method, it does not present a clear choice, *A*_1_ and *A*_2_ are acceptable. The enhanced approach produces more precise results.

#### 5.4.2 Comparison with TOPSIS method

The method of reference [129] is used to calculate, and the results are shown in Table 17.

**Table 17:** Results and ranking based on TOPSIS method.

Plan	*A*_1_	*A*_2_	*A*_3_	*A*_4_
**Comprehensive distance**	0.694782	0.639802	0.295253	0.282053
**Sort**	*A*_1_>*A*_2_>*A*_3_>*A*_4_			

Supplier *A*_1_ is the ideal provider, as shown in Table 17, which is in line with the findings of the paper construction model. However, the comprehensive distance between *A*_1_ and *A*_2_ that was found using the TOPSIS technique is quite modest, and the level of discrimination is only mildly present. In comparison, the VIKOR method’s evaluation is significant. The TOPSIS method’s guiding concept is separating the selected scheme from the positive and negative ideal solutions. The best option is not always the one closest to the ideal point based solely on distance measurements, and there is a reverse order problem brought on by the expansion or contraction of the design in actual use. This problem can be solved using the VIKOR approach. It is an ideal solution-based ranking compromise strategy. In this study, the multi-attribute decision-making problem is solved using the VIKOR approach based on neutrosophic sets to find a satisfying resolution. Instead of using the optimal solution, it opts for the ideal compromise.

#### 5.4.3 Comparison with weighted ensemble operator method

The method of reference [130] is used to calculate, and the results are shown in Table 18.

**Table 18:** Score function and ranking.

**Plan**	*A*_1_	*A*_2_	*A*_3_	*A*_4_
**Score**	0.6784	0.6630	0.5957	0.6134
**Rank**	*A*_1_>*A*_2_>*A*_4_>*A*_3_			

In the evaluation framework, the weighted operator is frequently applied and extended to single-valued neutrosophic sets. The accuracy of this strategy is confirmed by the ranking results’ consistency with the model’s output. The simple negative ideal solution distance is replaced by the relative distance in the model developed in this work instead. The candidate chosen by this method comes the closest to the ideal solution, and the findings are more accurate and reliable.

### 5.5 Discussion

This study suggests a sustainable supplier selection model and uses the MCDM method to put it into effect. Choosing the right MCDM techniques and supplier selection criteria are two fundamental concerns that assist businesses in managing product procurement and enhancing the entire supply chain. Considering the importance of human judgment and information provided by raw data, combining the consequences of AHP and target entropy to determine the criteria weight has proved to be one of the most effective methods.

Group and individual decision-making have always been critical issues and challenges in sustainable supplier selection; combining the advantages of both group and personal decision-making is wise. The method proposed in this paper provides a solution closer to ideal and a balance between minimal individual regret and maximum group utility. It is challenging for experts to accurately assess suppliers in practice since the environment for choosing sustainable suppliers is complex and unpredictable. The method suggested in this research considers the flawed information provided by managers, and decision-makers can try their best to communicate their evaluation information more precisely and efficiently by using SVNS, which offers an efficient method for choosing sustainable suppliers. Moreover, this method has lower complexity and is easier to apply to decision-making problems, saving decision-makers’ time and energy. The case study reviewed shows the model’s effectiveness, and relevant supply chain practitioners can make plans based on the results of supplier evaluation. Therefore, the results of this case can provide a reference for the development of a sustainable supply chain, which can save costs and reduce the negative impact brought by external factors.

Additionally, businesses are more likely to work with suppliers concerned about environmental issues due to external factors like policy and the environment. Therefore, while choosing suppliers, it is essential to be clear about the specifics of environmental management (such as pollution avoidance, resource consumption, discharge of harmful substances and wastes, etc.). Suppliers can take on social responsibility, check the legality of their production process, and confirm that primary and sub suppliers have signed environmental contracts to prevent potential environmental issues brought on by lower-level suppliers by signing legal agreements with them.

## 6 Conclusion

One of the modern businesses’ most significant challenges is sustainable supply chain management. Supplier selection is a crucial topic in engineering and management since an enterprise’s supply chain comprises several suppliers, and those suppliers’ qualifications impact the total benefit of the supply chain. To attain the objective of sustainable development, the corporation should prioritize social and environmental responsibility and maximize economic rewards.

The result of the study addressed the goals and objectives mentioned in the paper’s introduction. The first part of this study evaluates the literature on sustainable suppliers, quantifies and makes readily accessible a framework of environmental, social, and economic performance indicators, and analyses and formulates the selection criteria for sustainable suppliers. The total weight created by combining subjective and objective weights is used to indicate the importance of indicators, improving the ranking results’ effectiveness by taking into account the value of human judgment and the information provided by raw data. Additionally, the supplier selection issue is an MCDM issue.

Due to the constraints of the decision maker’s subjective cognition and the objective complexity of the actual decision-making problem, the decision-makers frequently communicate the assessment value of the scheme using fuzzy information. This study employs the notion of neutrosophic sets, SVNS to quantify ambiguous and inconsistent data, and the VIKOR method to analyze it. This study extends the VIKOR method using SVNS. The metric for SVNS distance substitutes the immediate difference of the original VIKOR method and introduces the idea of "relative distance," which makes the data processing more logical and scientific. This approach enhances the character of evaluation results by reducing the impact of individual preferences and experiences on the final evaluation results and the issue of particular excessive regret brought on by factor correlation. It also increases the consistency of the evaluation’s findings. Finally, the effectiveness and flexibility of the suggested method are confirmed through case analysis, sensitivity analysis, and comparative analysis of various forms. This method can effectively capture the competition among sustainable suppliers, offer more options for decision support systems, and better meet the needs of various practical problems.

The approach presented in this paper is very adaptable to sectors that support supply chain sustainability. Managers can use it to choose suitable suppliers to suit those sectors’ needs. In contrast to earlier studies, the index system developed in this study is more thorough based on the fundamental principles, and the suggested model is more appropriate for uncertain scenarios and nearer to human cognition. This model not only effectively manages information fuzziness and uncertainty but also maintains the accuracy of evaluation data. To assist decision-makers in expressing their ideas more convincingly and realistically in additional multi-attribute decision-making challenges, the developed SVNS model can be applied.

The study does have certain restrictions. Future research should consider the decision-making mechanism coefficient and any potential interactions and relationships between standards, as these elements have not been fully explored in the current study. Additionally, fuzzy and neutrosophic sets cannot fully capture fundamental uncertainty; therefore, future research will focus on combining multi-granularity language data with support vector machines, fuzzy sets, and neutrosophic sets to create a model known as the Multi-Criteria Decision Making (MCDM).

## References

[1] Xing Y, Cao M, Liu Y, Zhou M, Wu J. A Choquet integral based interval Type-2 trapezoidal fuzzy multiple attribute group decision making for Sustainable Supplier Selection. *Computers & Industrial Engineering*. 2022;165: 107935. 10.1016/j.cie.2022.107935.

[2] Cikovic KF, Martincevic I, Lozic J. Application of Data Envelopment Analysis (DEA) in the Selection of Sustainable Suppliers: A Review and Bibliometric Analysis. *Sustainability*. 2022;14(11): 6672. 10.3390/su14116672.

[3] Finger GSW, Lima-Junior FR. A hesitant fuzzy linguistic QFD approach for formulating sustainable supplier development programs. *International Journal of Production Economics*. 2022;247: 108428. 10.1016/j.ijpe.2022.108428.

[4] Fan D, Xiao C, Zhang X, Guo Y. Gaining customer satisfaction through sustainable supplier development: The role of firm reputation and marketing communication. *Transportation Research Part E-Logistics and Transportation Review*. 2021;154: 102453. 10.1016/j.tre.2021.102453.

[5] Yan X, Bao X, Zhao R, Li F. Performance measurement for green supplier selection based on data envelopment analysis. *Environmental Science and Pollution Research*. 2022;29(30): 45960–45970. doi: 10.1007/s11356-021-17897-2 35156165

[6] Mishra AR, Saha A, Rani P, Pamucar D, Dutta D, Hezam IM. Sustainable supplier selection using HF-DEA-FOCUM-MABAC technique: a case study in the Auto-making industry. *Soft Computing*. 2022;26(17): 8821–8840. doi: 10.1007/s00500-022-07192-8 35677555 PMC9164192

[7] Bai C, Satir A. A critical content-analysis of sustainable supplier development literature and future research directions. *Journal of Cleaner Production*. 2022;365: 132443. 10.1016/j.jclepro.2022.132443.

[8] Shang Z, Yang X, Barnes D, Wu C. Supplier selection in sustainable supply chains: Using the integrated BWM, fuzzy Shannon entropy, and fuzzy MULTIMOORA methods. *Expert Systems with Applications*. 2022;195: 116567. 10.1016/j.eswa.2022.116567.

[9] Sinha AK, Anand A. Development of sustainable supplier selection index for new product development using multi criteria decision making. *Journal of Cleaner Production*. 2018;197: 1587–1596. 10.1016/j.jclepro.2018.06.234.

[10] Xu D, Cui X, Xian H. An Extended EDAS Method with a Single-Valued Complex Neutrosophic Set and Its Application in Green Supplier Selection. *Mathematics*. 2020;8(2): 282. 10.3390/math8020282.

[11] Kaur P, Dutta V, Pradhan BL, Haldar S, Chauhan S. A Pythagorean Fuzzy Approach for Sustainable Supplier Selection Using TODIM. *Mathematical Problems in Engineering*. 2021;2021: 4254894. 10.1155/2021/4254894.

[12] Rashidi K, Noorizadeh A, Kannan D, Cullinane K. Applying the triple bottom line in sustainable supplier selection: A meta-review of the state-of-the-art. *Journal of Cleaner Production*. 2020;269: 122001. 10.1016/j.jclepro.2020.122001.

[13] Rajesh R, Ravi V. Supplier selection in resilient supply chains: a grey relational analysis approach. *Journal of Cleaner Production*. 2015;86: 343–359. 10.1016/j.jclepro.2014.08.054.

[14] Unal Y, Temur GT. Sustainable supplier selection by using spherical fuzzy AHP. *Journal of Intelligent & Fuzzy Systems*. 2022;42(1): 593–603. 10.3233/JIFS-219214.

[15] Zakeri S, Yang Y, Konstantas D. A Supplier Selection Model Using Alternative Ranking Process by Alternatives’ Stability Scores and the Grey Equilibrium Product. *Processes*. 2022;10(5): 917. 10.3390/pr10050917.

[16] Aslani B, Rabiee M, Tavana M. An integrated information fusion and grey multi-criteria decision-making framework for sustainable supplier selection. *International Journal of Systems Science-Operations &* *Logistics*. 2021;8(4): 348–370. 10.1080/23302674.2020.1776414.

[17] Laurin F, Fantazy K. Sustainable supply chain management: a case study at IKEA. *Transnational Corporations Review*. 2017;9(4): 309–318. 10.1080/19186444.2017.1401208.

[18] Masoomi B, Fathi M, Yildirim F, Ghorbani S, Sahebi IG. Strategic supplier selection for renewable energy supply chain under green capabilities (fuzzy BWM-WASPAS-COPRAS approach). *Energy Strategy Reviews*. 2022;40: 100815. 10.1016/j.esr.2022.100815.

[19] Giri BC, Molla MU, Biswas P. Pythagorean fuzzy DEMATEL method for supplier selection in sustainable supply chain management. *Expert Systems with Applications*. 2022;193: 116396. 10.1016/j.eswa.2021.116396.

[20] Chen SM, Han WH. An improved MADM method using interval-valued intuitionistic fuzzy values. *Information Sciences*. 2018;467: 489–505. 10.1016/j.ins.2018.07.062.

[21] Ghadimi P, Toosi FG, Heavey C. A multi-agent systems approach for sustainable supplier selection and order allocation in a partnership supply chain. *European Journal of Operational Research*. 2018;269(1): 286–301. 10.1016/j.ejor.2017.07.014.

[22] Chai J, Ngai EWT. Decision-making techniques in supplier selection: Recent accomplishments and what lies ahead. *Expert Systems with Applications*. 2020;140: 112903. 10.1016/j.eswa.2019.112903.

[23] Azadi M, Jafarian M, Saen RF, Mirhedayatian SM. A new fuzzy DEA model for evaluation of efficiency and effectiveness of suppliers in sustainable supply chain management context. *Computers & Operations Research*. 2015;54: 274–285. 10.1016/j.cor.2014.03.002.

[24] Keshavarz Ghorabaee M, Amiri M, Zavadskas EK, Turskis Z, Antucheviciene J. A new multi-criteria model based on interval type-2 fuzzy sets and EDAS method for supplier evaluation and order allocation with environmental considerations. *Computers & Industrial Engineering*. 2017;112: 156–174. 10.1016/j.cie.2017.08.017.

[25] Demir L, Akpinar ME, Araz C, Ilgin MA. A green supplier evaluation system based on a new multi-criteria sorting method: VIKORSORT. *Expert Systems with Applications*. 2018;114: 479–487. 10.1016/j.eswa.2018.07.071.

[26] Ecer F, Torkayesh AE. A Stratified Fuzzy Decision-Making Approach for Sustainable Circular Supplier Selection. *Ieee Transactions on Engineering Management*. 10.1109/TEM.2022.3151491.

[27] Seuring S, Mueller M. From a literature review to a conceptual framework for sustainable supply chain management. *Journal of Cleaner Production*. 2008;16(15): 1699–1710. 10.1016/j.jclepro.2008.04.020.

[28] Coskun SS, Kumru M, Kan NM. An integrated framework for sustainable supplier development through supplier evaluation based on sustainability indicators. *Journal of Cleaner Production*. 2022;335: 130287. 10.1016/j.jclepro.2021.130287.

[29] Oh J, Jeong B. Tactical supply planning in smart manufacturing supply chain. *Robotics and Computer-Integrated Manufacturing*. 2019;55: 217–233. 10.1016/j.rcim.2018.04.003.

[30] Albrecht W, Steinruecke M. Coordinating continuous-time distribution and sales planning of perishable goods with quality grades. *International Journal of Production Research*. 2018;56(7): 2646–2665. 10.1080/00207543.2017.1384584.

[31] Margolis JT, Sullivan KM, Mason SJ, Magagnotti M. A multi-objective optimization model for designing resilient supply chain networks. *International Journal of Production Economics*. 2018;204: 174–185. 10.1016/j.ijpe.2018.06.008.

[32] Muhammad N, Fang Z, Shah SAA, Akbar MA, Alsanad A, Gumaei A, et al. A Hybrid Multi-Criteria Approach for Evaluation and Selection of Sustainable Suppliers in the Avionics Industry of Pakistan. *Sustainability*. 2020;12(11): 4744. 10.3390/su12114744.

[33] Fahimnia B, Sarkis J, Eshragh A. A tradeoff model for green supply chain planning: A leanness-versus-greenness analysis. *Omega-International Journal of Management Science*. 2015;54: 173–190. 10.1016/j.omega.2015.01.014.

[34] Saxena LK, Jain PK, Sharma AK. Tactical supply chain planning for tyre remanufacturing considering carbon tax policy. *International Journal of Advanced Manufacturing Technology*. 2018;97(1–4): 1505–1528. 10.1007/s00170-018-1972-3.

[35] Zarbakhshnia N, Soleimani H, Goh M, Razavi SS. A novel multi-objective model for green forward and reverse logistics network design. *Journal of Cleaner Production*. 2019;208: 1304–1316. 10.1016/j.jclepro.2018.10.138.

[36] Boukherroub T, Ruiz A, Guinet A, Fondrevelle J. An integrated approach for sustainable supply chain planning. *Computers & Operations Research*. 2015;54: 180–194. 10.1016/j.cor.2014.09.002.

[37] Sotoudeh-Anvari A. The applications of MCDM methods in COVID-19 pandemic: A state of the art review. *Applied Soft Computing*. 2022;126: 109238. doi: 10.1016/j.asoc.2022.109238 35795407 PMC9245376

[38] Yin C, Ji F, Weng X, Zhang Q, Geng S. The optimal plan selection framework of rail transit photovoltaic power station under probabilistic linguistic environment. *Journal of Cleaner Production*. 2021;328: 129560. 10.1016/j.jclepro.2021.129560.

[39] Ayyildiz E, Taskin A. A novel spherical fuzzy AHP-VIKOR methodology to determine serving petrol station selection during COVID-19 lockdown: A pilot study for İstanbul. *Socio-Economic Planning Sciences*. 2022;83: 101345. 10.1016/j.seps.2022.101345. 35645424 PMC9126831

[40] Wang Z, Ran Y, Chen Y, Yu H, Zhang G. Failure mode and effects analysis using extended matter-element model and AHP. *Computers & Industrial Engineering*. 2020;140: 106233. 10.1016/j.cie.2019.106233.

[41] Govindan K, Rajendran S, Sarkis J, Murugesan P. Multi criteria decision making approaches for green supplier evaluation and selection: a literature review. *Journal of Cleaner Production*. 2015;98: 66–83. 10.1016/j.jclepro.2013.06.046.

[42] Ma W, Du Y, Liu X, Shen Y. Literature review: Multi-criteria decision-making method application for sustainable deep-sea mining transport plans. *Ecological Indicators*. 2022;140: 109049. 10.1016/j.ecolind.2022.109049.

[43] Park K, Kremer GEO, Ma J. A regional information-based multi-attribute and multi-objective decision-making approach for sustainable supplier selection and order allocation. *Journal of Cleaner Production*. 2018;187: 590–604. 10.1016/j.jclepro.2018.03.035.

[44] Dweiri F, Kumar S, Khan SA, Jain V. Designing an integrated AHP based decision support system for supplier selection in automotive industry. *Expert Systems with Applications*. 2016;62: 273–283. 10.1016/j.eswa.2016.06.030.

[45] Xie N, Xin J. Interval grey numbers based multi-attribute decision making method for supplier selection. *Kybernetes*. 2014;43(7): 1064–1078. 10.1108/K-01-2014-0010.

[46] Akram M, Garg H, Zahid K. Extensions of ELECTRE-I and TOPSIS methods for group decision-making under complex Pythagorean fuzzy environment. *Iranian Journal of Fuzzy Systems*. 2020;17(5): 147–164.

[47] Ahmadi O, Mortazavi SB, Mahabadi HA, Hosseinpouri M. Development of a dynamic quantitative risk assessment methodology using fuzzy DEMATEL-BN and leading indicators. *Process Safety and Environmental Protection*. 2020;142: 15–44. 10.1016/j.psep.2020.04.038.

[48] Ullah K, Mahmood T, Ali Z, Jan N. On some distance measures of complex Pythagorean fuzzy sets and their applications in pattern recognition. *Complex & Intelligent Systems*. 2020;6(1): 15–27. 10.1007/s40747-019-0103-6.

[49] Sarma D, Das A, Dutta P, Bera UK. A Cost Minimization Resource Allocation Model for Disaster Relief Operations With an Information Crowdsourcing-Based MCDM Approach. *Ieee Transactions on Engineering Management*. 2022;69(5): 2454–2474. 10.1109/TEM.2020.3015775.

[50] Singh A, Majumder P, Bera UK. Prediction of polypropylene business strategy for a petrochemical plant using a technique for order preference by similarity to an ideal solution-based artificial neural network. *Polymer Engineering and Science*. 2022;62(4): 1096–1113. doi: 10.1002/pen.25909

[51] Hosseini SM, Paydar MM, Triki C. Implementing sustainable ecotourism in Lafour region, Iran: Applying a clustering method based on SWOT analysis. *Journal of Cleaner Production*. 2021;329: 129716. 10.1016/j.jclepro.2021.129716.

[52] Kaya I, Colak M, Terzi F. A comprehensive review of fuzzy multi criteria decision making methodologies for energy policy making. *Energy Strategy Reviews*. 2019;24: 207–228. 10.1016/j.esr.2019.03.003.

[53] Xiao J, Cai M, Gao Y. A VIKOR-Based Linguistic Multi-Attribute Group Decision-Making Model in a Quantum Decision Scenario. *Mathematics*. 2022;10(13): 2236. 10.3390/math10132236.

[54] Zhang H, Wei G, Chen X. SF-GRA method based on cumulative prospect theory for multiple attribute group decision making and its application to emergency supplies supplier selection. *Engineering Applications of Artificial Intelligence*. 2022;110: 104679. 10.1016/j.engappai.2022.104679.

[55] Thanh NV, Lan NTK. A New Hybrid Triple Bottom Line Metrics and Fuzzy MCDM Model: Sustainable Supplier Selection in the Food-Processing Industry. *Axioms*. 2022;11(2): 57. 10.3390/axioms11020057.

[56] Liu A, Xiao Y, Lu H, Tsai SB, Song W. A fuzzy three-stage multi-attribute decision-making approach based on customer needs for sustainable supplier selection. *Journal of Cleaner Production*. 2019;239: 118043. 10.1016/j.jclepro.2019.118043.

[57] wen Shen K, kang Wang X, Qiao D, qiang Wang J. Extended Z-MABAC Method Based on Regret Theory and Directed Distance for Regional Circular Economy Development Program Selection With Z-Information. *Ieee Transactions on Fuzzy Systems*. 2020;28(8): 1851–1863. 10.1109/TFUZZ.2019.2923948.

[58] Zadeh LA. Fuzzy Sets. *Information & Control*. 1965;8(3): 338–353.

[59] Akram M, Adeel A, Al-Kenani AN, Alcantud JCR. Hesitant fuzzy N-soft ELECTRE-II model: a new framework for decision-making. *Neural Computing & Applications*. 2021;33(13): 7505–7520. 10.1007/s00521-020-05498-y.

[60] Chai J, Liu JNK, Ngai EWT. Application of decision-making techniques in supplier selection: A systematic review of literature. *Expert Systems with Applications*. 2013;40(10): 3872–3885. 10.1016/j.eswa.2012.12.040.

[61] Diaz-Vazquez S, Torres-Manzanera E, Diaz I, Montes S. On the Search for a Measure to Compare Interval-Valued Fuzzy Sets. *Mathematics*. 2021;9(24): 3157. 10.3390/math9243157.

[62] Niu X, Sun Z, Kong X. A new type of dyad fuzzy beta-covering rough set models base on fuzzy information system and its practical application. *International Journal of Approximate Reasoning*. 2022;142: 13–30. 10.1016/j.ijar.2021.11.001.

[63] Atanassov KT. Intuitionistic fuzzy sets. *Fuzzy Sets and Systems*. 1986;20(1): 87–96. 10.1016/S0165-0114(86)80034-3.

[64] Atanassov K, Gargov G. Interval valued intuitionistic fuzzy sets. *Fuzzy Sets and Systems*. 1989;31(3): 343–349. 10.1016/0165-0114(89)90205-4.

[65] John R. Fuzzy Sets of Type-2. *Journal of Advanced Computational Intelligence and Intelligent Informatics*. 1999;3(6): 499–508. 10.20965/jaciii.1999.p0499.

[66] Lv ZH, Chen CB, Qiu PY. Normal Distribution Fuzzy Sets A New Extension of Fuzzy Sets. 11. 2006; 1–4.

[67] Torra V. Hesitant Fuzzy Sets. *International Journal of Intelligent Systems*. 2010;25(6): 529–539. 10.1002/int.20418.

[68] Jain N, Singh AR. Sustainable supplier selection under must-be criteria through Fuzzy inference system. *Journal of Cleaner Production*. 2020;248: 119275. 10.1016/j.jclepro.2019.119275.

[69] Akram M, Kahraman C, Zahid K. Extension of TOPSIS model to the decision-making under complex spherical fuzzy information. *Soft Computing*. 2021;25(16): 10771–10795. 10.1007/s00500-021-05945-5.

[70] Smarandache F. A unifying field in logics: Neutrosophic logic. *Multiple-Valued Logic*. 1999;8(3): 1–153.

[71] Wang H, Smarandache F, Zhang YQ, Sunderraman R. Interval Neutrosophic Sets and Logic: Theory and Applications in Computing. *Computing Research Repository—CORR*. 2005. 10.5281/zenodo.8818.

[72] Ye J. A multicriteria decision-making method using aggregation operators for simplified neutrosophic sets. *Journal of Intelligent & Fuzzy Systems*. 2014;26(5): 2459–2466. 10.3233/IFS-130916.

[73] Yazdani M, Torkayesh AE, Stevic Z, Chatterjee P, Ahari SA, Hernandez VD. An interval valued neutrosophic decision-making structure for sustainable supplier selection. *Expert Systems with Applications*. 2021;183: 115354. 10.1016/j.eswa.2021.115354.

[74] Vafadarnikjoo A, Ahmadi HB, Liou JJH, Botelho T, Chalvatzis K. Analyzing blockchain adoption barriers in manufacturing supply chains by the neutrosophic analytic hierarchy process. *Annals of Operations Research*. 10.1007/s10479-021-04048-6.

[75] Abdel-Basset M, Gamal A, Chakrabortty RK, Ryan M. Development of a hybrid multi-criteria decision-making approach for sustainability evaluation of bioenergy production technologies: A case study. *Journal of Cleaner Production*. 2021;290: 125805. 10.1016/j.jclepro.2021.125805.

[76] Rani P, Mishra AR, Krishankumar R, Ravichandran KS, Kar S. Multi-criteria food waste treatment method selection using single-valued neutrosophic-CRITIC-MULTIMOORA framework. *Applied Soft Computing*. 2021;111: 107657. 10.1016/j.asoc.2021.107657. PMC7862040 33568967

[77] Abdel-Basset M, Manogaran G, Gamal A, Smarandache F. A Group Decision Making Framework Based on Neutrosophic TOPSIS Approach for Smart Medical Device Selection. *Journal of Medical Systems*. 2019;43(2): 38. doi: 10.1007/s10916-019-1156-1 30627801

[78] Nafei A, Javadpour A, Nasseri H, Yuan W. Optimized score function and its application in group multiattribute decision making based on fuzzy neutrosophic sets. *International Journal of Intelligent Systems*. 2021;36(12): 7522–7543. 10.1002/int.22597.

[79] Opricovic S. Multicriteria optimization of civil engineering systems. *Faculty of Civil Engineering*. 1998;37: 5–21.

[80] Jianxing Y, Shibo W, Haicheng C, Yang Y, Haizhao F, Jiahao L. Risk assessment of submarine pipelines using modified FMEA approach based on cloud model and extended VIKOR method. *Process Safety and Environmental Protection*. 2021;155: 555–574. doi: 10.1016/j.psep.2021.09.047

[81] Hadian H, Chahardoli S, Golmohammadi AM, Mostafaeipour A. A practical framework for supplier selection decisions with an application to the automotive sector. *International Journal of Production Research*. 2020;58(10): 2997–3014. 10.1080/00207543.2019.1624854.

[82] Wang C, Li J. Project investment decision based on VIKOR interval intuitionistic fuzzy set. *Journal of Intelligent & Fuzzy Systems*. 2022;42(2): 623–631. 10.3233/JIFS-189735.

[83] Ansari ZN, Kant R, Shankar R. Evaluation and ranking of solutions to mitigate sustainable remanufacturing supply chain risks: a hybrid fuzzy SWARA-fuzzy COPRAS framework approach. *International Journal of Sustainable Engineering*. 2020;13(6): 473–494. 10.1080/19397038.2020.1758973.

[84] Ma Y, Guga S, Xu J, Liu X, Tong Z, Zhang J. Assessment of Maize Drought Risk in Midwestern Jilin Province: A Comparative Analysis of TOPSIS and VIKOR Models. *Remote Sensing*. 2022;14(10): 2399. 10.3390/rs14102399.

[85] Opricovic S, Tzeng GH. Compromise solution by MCDM methods: A comparative analysis of VIKOR and TOPSIS. *European Journal of Operational Research*. 2004;156(2): 445–455. 10.1016/s0377-2217(03)00020-1.

[86] Wu C, Lin Y, Barnes D. An integrated decision-making approach for sustainable supplier selection in the chemical industry. *Expert Systems with Applications*. 2021;184: 115553. 10.1016/j.eswa.2021.115553.

[87] Huang H L. New Distance Measure of Single-valued Neutrosophic Sets and Its Application. *International Journal of Intelligent Systems*, 2016, 31: 1021–1032.

[88] Li DP, Xie L, Cheng PF, Zhou XH, Fu CX. Green Supplier Selection Under Cloud Manufacturing Environment: A Hybrid MCDM Model. *Sage Open*. 2021;11(4): 21582440211057110. 10.1177/21582440211057112.

[89] Ecer F, Pamucar D. Sustainable supplier selection: A novel integrated fuzzy best worst method (F-BWM) and fuzzy CoCoSo with Bonferroni (CoCoSo’B) multi-criteria model. *Journal of Cleaner Production*. 2020;266: 121981. 10.1016/j.jclepro.2020.121981.

[90] Rashidi K, Cullinane K. A comparison of fuzzy DEA and fuzzy TOPSIS in sustainable supplier selection: Implications for sourcing strategy. *Expert Systems with Applications*. 2019;121: 266–281. 10.1016/j.eswa.2018.12.025.

[91] Yu C, Shao Y, Wang K, Zhang L. A group decision making sustainable supplier selection approach using extended TOPSIS under interval-valued Pythagorean fuzzy environment. *Expert Systems with Applications*. 2019;121: 1–17. 10.1016/j.eswa.2018.12.010.

[92] Phochanikorn P, Tan C. A New Extension to a Multi-Criteria Decision-Making Model for Sustainable Supplier Selection under an Intuitionistic Fuzzy Environment. *Sustainability*. 2019;11(19): 5413. 10.3390/su11195413.

[93] Awasthi A, Govindan K, Gold S. Multi-tier sustainable global supplier selection using a fuzzy AHP-VIKOR based approach. *International Journal of Production Economics*. 2018;195: 106–117. 10.1016/j.ijpe.2017.10.013.

[94] Zhang J, Yang D, Li Q, Lev B, Ma Y. Research on Sustainable Supplier Selection Based on the Rough DEMATEL and FVIKOR Methods. *Sustainability*. 2021;13(1): 88. 10.3390/su13010088.

[95] Peng J juan, Tian C, Zhang W yu, Zhang S, qiang Wang J. An Integrated Multi-Criteria Decision-Making Framework for Sustainable Supplier Selection Under Picture Fuzzy Environment. *Technological and Economic Development of Economy*. 2020;26(3): 573–598. 10.3846/tede.2020.12110.

[96] Alrasheedi M, Mardani A, Mishra AR, Rani P, Loganathan N. An extended framework to evaluate sustainable suppliers in manufacturing companies using a new Pythagorean fuzzy entropy-SWARA-WASPAS decision-making approach. *Journal of Enterprise Information Management*. 2022;35(2): 333–357. 10.1108/JEIM-07-2020-0263.

[97] Puška A, Nedeljković M, Hashemkhani Zolfani S, Pamučar D. Application of Interval Fuzzy Logic in Selecting a Sustainable Supplier on the Example of Agricultural Production. *Symmetry*. 2021;13(5): 774. 10.3390/sym13050774.

[98] Zhang J, Li L, Zhang J, Chen L, Chen G. Private-label sustainable supplier selection using a fuzzy entropy-VIKOR-based approach. *Complex & Intelligent Systems*. 10.1007/s40747-021-00317-w.

[99] Memari A, Dargi A, Akbari Jokar MR, Ahmad R, Abdul Rahim AbdR. Sustainable supplier selection: A multi-criteria intuitionistic fuzzy TOPSIS method. *Journal of Manufacturing Systems*. 2019;50: 9–24. 10.1016/j.jmsy.2018.11.002.

[100] Van LH, Yu VF, Dat LQ, Dung CC, Chou SY, Loc NV. New Integrated Quality Function Deployment Approach Based on Interval Neutrosophic Set for Green Supplier Evaluation and Selection. *Sustainability*. 2018;10(3): 838. 10.3390/su10030838.

[101] Xu H, Chen L, Li Q, Yang J. A Multi-Attribute Decision Method under Uncertainty Environment Conditions-The Green Supplier Evaluation Perspective. *International Journal of Environmental Research and Public Health*. 2021;18(1): 344. doi: 10.3390/ijerph18010344 33466386 PMC7796048

[102] Chang TW, Pai CJ, Lo HW, Hu SK. A hybrid decision-making model for sustainable supplier evaluation in electronics manufacturing. *Computers & Industrial Engineering*. 2021;156: 107283. 10.1016/j.cie.2021.107283.

[103] Nguyen NBT, Lin GH, Dang TT. A Two Phase Integrated Fuzzy Decision-Making Framework for Green Supplier Selection in the Coffee Bean Supply Chain. *Mathematics*. 2021;9(16): 1923. 10.3390/math9161923.

[104] Yucesan M, Mete S, Serin F, Celik E, Gul M. An Integrated Best-Worst and Interval Type-2 Fuzzy TOPSIS Methodology for Green Supplier Selection. *Mathematics*. 2019;7(2): 182. 10.3390/math7020182.

[105] Xu Z, Qin J, Liu J, Martínez L. Sustainable supplier selection based on AHPSort II in interval type-2 fuzzy environment. *Information Sciences*. 2019;483: 273–293. 10.1016/j.ins.2019.01.013.

[106] Osiro L, Costa RA de MBV da, Lima Junior FR. Evaluating supplier sustainability using fuzzy 2-tuple representation. *Gestão & Produção*. 2021;28(1): e4933. 10.1590/1806-9649.2020v28e4933.

[107] Rabbani M, Foroozesh N, Mousavi SM, Farrokhi-Asl H. Sustainable supplier selection by a new decision model based on interval-valued fuzzy sets and possibilistic statistical reference point systems under uncertainty. *International Journal of Systems Science-Operations & Logistics*. 2019;6(2): 162–178. 10.1080/23302674.2017.1376232.

[108] Ecer F. Multi-criteria decision making for green supplier selection using interval type-2 fuzzy AHP: a case study of a home appliance manufacturer. *Operational Research*. 2022;22(1): 199–233. 10.1007/s12351-020-00552-y.

[109] Kannan D. Role of multiple stakeholders and the critical success factor theory for the sustainable supplier selection process. *International Journal of Production Economics*. 2018;195: 391–418. 10.1016/j.ijpe.2017.02.020.

[110] Hoseini SA, Fallahpour A, Wong KY, Mahdiyar A, Saberi M, Durdyev S. Sustainable Supplier Selection in Construction Industry through Hybrid Fuzzy-Based Approaches. *Sustainability*. 2021;13(3): 1413. 10.3390/su13031413.

[111] Pinar A, Boran FE. A q-rung orthopair fuzzy multi-criteria group decision making method for supplier selection based on a novel distance measure. *International Journal of Machine Learning and Cybernetics*. 2020;11(8): 1749–1780. 10.1007/s13042-020-01070-1.

[112] Alikhani R, Torabi SA, Altay N. Strategic supplier selection under sustainability and risk criteria. *International Journal of Production Economics*. 2019;208: 69–82. 10.1016/j.ijpe.2018.11.018.

[113] Li J, Fang H, Song W. Sustainable supplier selection based on SSCM practices: A rough cloud TOPSIS approach. *Journal of Cleaner Production*. 2019;222: 606–621. 10.1016/j.jclepro.2019.03.070.

[114] Tong L, Pu Z, Chen K, Yi J. Sustainable maintenance supplier performance evaluation based on an extend fuzzy PROMETHEE II approach in petrochemical industry. *Journal of Cleaner Production*. 2020;273: 122771. 10.1016/j.jclepro.2020.122771.

[115] Liu C, Rani P, Pachori K. Sustainable circular supplier selection and evaluation in the manufacturing sector using Pythagorean fuzzy EDAS approach. *Journal of Enterprise Information Management*. 2022;35(4/5): 1040–1066. 10.1108/JEIM-04-2021-0187.

[116] Song Y, Wang J, Guo F, Lu J, Liu S. Research on Supplier Selection of Prefabricated Building Elements from the Perspective of Sustainable Development. *Sustainability*. 2021;13(11): 6080. 10.3390/su13116080.

[117] ping Wan S, chang Zou W, gen Zhong L, ying Dong J. Some new information measures for hesitant fuzzy PROMETHEE method and application to green supplier selection. *Soft Computing*. 2020;24(12): 9179–9203. 10.1007/s00500-019-04446-w.

[118] Hendiani S, Mahmoudi A, Liao H. A multi-stage multi-criteria hierarchical decision-making approach for sustainable supplier selection. *Applied Soft Computing*. 2020;94: 106456. 10.1016/j.asoc.2020.106456.

[119] Wang X, Wang H, Xu Z, Ren Z. Green supplier selection based on probabilistic dual hesitant fuzzy sets: A process integrating best worst method and superiority and inferiority ranking. *Applied Intelligence*. 2022;52(7): 8279–8301. 10.1007/s10489-021-02821-5.

[120] Foroozesh N, Jolai F, Mousavi SM, Karimi B. A new fuzzy-stochastic compromise ratio approach for green supplier selection problem with interval-valued possibilistic statistical information. *Neural Computing & Applications*. 2021;33(13): 7893–7911. 10.1007/s00521-020-05527-w.

[121] Calik A. A hybrid approach for selecting sustainable suppliers and determining order allocation based on interval type-2 fuzzy sets. *Journal of Enterprise Information Management*. 2020;33(5): 923–945. 10.1108/JEIM-09-2019-0302.

[122] Bai C, Kusi-Sarpong S, Badri Ahmadi H, Sarkis J. Social sustainable supplier evaluation and selection: a group decision-support approach. *International Journal of Production Research*. 2019;57(22): 7046–7067. 10.1080/00207543.2019.1574042.

[123] Abdel-Basset M, Mohamed M, Smarandache F. A Hybrid Neutrosophic Group ANP-TOPSIS Framework for Supplier Selection Problems. *Symmetry-Basel*. 2018;10(6): 226. 10.3390/sym10060226.

[124] Saaty TL. A scaling method for priorities in hierarchical structures. *Journal of Mathematical Psychology*. 1977;15(3): 234–281. 10.1016/0022-2496(77)90033-5.

[125] Saaty TL. Relative Measurement and Its Generalization in Decision Making Why Pairwise Comparisons are Central in Mathematics for the Measurement of Intangible Factors The Analytic Hierarchy/Network Process (To the Memory of my Beloved Friend Professor Sixto Rios Garcia). *Revista De La Real Academia De Ciencias Exactas Fisicas Y Naturales Serie a-Matematicas*. 2008;102(2): 251–318. 10.1007/BF03191825.

[126] Zhang D, Zhao M, Wei G, Chen X. Single-valued neutrosophic TODIM method based on cumulative prospect theory for multi-attribute group decision making and its application to medical emergency management evaluation. Economic Research-Ekonomska Istrazivanja. 10.1080/1331677X.2021.2013914.

[127] Zhong Y, Wang H, Lv H, Guo F. A vertical handoff decision scheme using subjective-objective weighting and grey relational analysis in cognitive heterogeneous networks. *Ad Hoc Networks*. 2022;134: 102924. 10.1016/j.adhoc.2022.102924.

[128] Wang X, Geng Y, Yao P, Yang M. Multiple attribute group decision making approach based on extended VIKOR and linguistic neutrosophic Set. *Journal of Intelligent & Fuzzy Systems*. 2019;36(1): 149–160. 10.3233/JIFS-181066.

[129] Azimifard A, Moosavirad SH, Ariafar S. Selecting sustainable supplier countries for Iran’s steel industry at three levels by using AHP and TOPSIS methods. *Resources Policy*. 2018;57: 30–44. 10.1016/j.resourpol.2018.01.002.

[130] Mishra AR, Rani P, Prajapati RS. Multi-criteria weighted aggregated sum product assessment method for sustainable biomass crop selection problem using single-valued neutrosophic sets. *Applied Soft Computing*. 2021;113: 108038. 10.1016/j.asoc.2021.108038.

[131] Xu DS, Wei C, Wei GW. TODIM Method for Single-Valued Neutrosophic Multiple Attribute Decision Making. *Information*. 2017;8(4): 125. 10.3390/info8040125.

[132] Eroglu H, Sahin R. A Neutrosophic VIKOR Method-Based Decision-Making with an Improved Distance Measure and Score Function: Case Study of Selection for Renewable Energy Alternatives. *Cognitive Computation*. 2020;12(6): 1338–1355. 10.1007/s12559-020-09765-x.

